# Visual outcome of endogenous endophthalmitis in Thailand

**DOI:** 10.1038/s41598-021-93730-7

**Published:** 2021-07-12

**Authors:** Preeyachan Lourthai, Pitipol Choopong, Dhanach Dhirachaikulpanich, Kunravitch Soraprajum, Warinyupa Pinitpuwadol, Noppakhun Punyayingyong, Yaninsiri Ngathaweesuk, Nattaporn Tesavibul, Sutasinee Boonsopon

**Affiliations:** 1 Ophthalmology Department, Mettapracharak Hospital, Rai Khing, Sampran, Nakornpathom, 73210 Thailand; 2 Department of Ophthalmology, Faculty of Medicine, Siriraj Hospital, Mahidol University, 2 Wanglang Road, Bangkoknoi, Bangkok, 10700 Thailand; 3 Department of Ophthalmology, Faculty of Medicine, Phramongkutklao Hospital, Phramongkutklao College of Medicine, 315 Ratchawithi Road, Thung Payathai, Ratchathewi, Bangkok, 10400 Thailand

**Keywords:** Retinal diseases, Uveal diseases

## Abstract

To evaluate a 10-year visual outcome of endogenous endophthalmitis (EE) patients. A 10-year retrospective chart review of EE patients. Thirty-eight patients (40 eyes) were diagnosed with EE at the mean age of 42. Among the identifiable pathogens (71.1% culture positive), the causative agents were predominantly gram-negative bacteria (48.1%). The most common specie was *Klebsiella pneumoniae* (25.9%). About a quarter of the patients required surgical eye removal, and the remaining 45.7% had visual acuity (VA) worse than hand motion at one month after the infectious episode. The most common complication was ocular hypertension (52.5%). Poor initial VA was significantly associated with a worse visual outcome in the early post-treatment period (*p* 0.12, adjusted OR 10.20, 95% CI 1.65–62.96). Five patients continued to visit the clinic for at least ten years. One patient had gained his vision from hand motion to 6/7.5. Two patients had visual deterioration, one from corneal decompensation, and the other from chronic retinal re-detachment. Two patients developed phthisis bulbi, with either some VA perception of light or no light perception. Poor initial VA is the only prognostic factor of a poor early post-treatment visual outcome of EE.

## Introduction

Endogenous endophthalmitis (EE) is a rare sight-threatening condition caused by the hematogenous seeding of microorganisms. The incidence rates are 0.04–0.4%. Risk factors include diabetes mellitus, intravenous drug use, immunocompromise, chronic systemic illness, and malignancy^1,2^. Causative pathogens, the origin of infections, and the clinical characteristics vary with the demographic region. Gram-negative bacterial endophthalmitis, especially *Klebsiella pneumonia*, is common in East Asia. In contrast, gram-positive bacteria predominate in Western countries^2–5^. The visual outcome is mostly poor. The factors associated with a worse prognosis include pathogen virulence, delayed treatment, and compromised host conditions^2,3^.

Most studies of EE have reported a short-term visual outcome—mainly, immediately post-treatment or after short follow-up periods. Over time, after the infection has subsided, complications might continue to develop. Some complications are reversible, but some may cause permanent ocular damage. Previous reports rarely showed the long-term consequences after the treatment. Thus, we aim to evaluate the long-term visual outcome and complications ten years after developing EE. We also describe the individuals’ initial clinical presentations, microbiology spectrums, treatments, and prognostic factors vis a vis a poor initial visual outcome.

## Materials and methods

A retrospective chart review of patients diagnosed with EE between September 2001 and August 2011 at Siriraj Hospital, Mahidol University was performed. EE was diagnosed based on a decrease in visual acuity (VA) with or without ocular pain, and panuveitis with a suspected history of recent, prior, or concurrent systemic infection. The patients with potential exogenous causes such as previous recent ocular surgery, ocular trauma, or a corneal ulcer were excluded from the study. Collected data includes demographics, associated systemic and ocular conditions, initial clinical features, systemic infection, microbiological profiles, treatment modalities, the final visual outcome, and any complications. We generally reported VA in meters. VA was converted to LogMAR only when a statistical calculation was necessary. Standard visual conversion was used from 6/6 to hand motion (HM) to 0-to-2.3 LogMAR^6^. VA of projection or perception of light as 3.7, and no light perception as 4.7, simply for calculation purposes, as it was previously used by Tsai et al.^7^. Statistical analyses included descriptive statistics, Chi-square and Fischer’s exact test was performed in a univariate analysis. Significant variables in a univariate analysis were then analyzed using the multivariate logistic-regression model. All analyses were performed using SPSS Statistics version 18.0 (SPSS Inc., Chicago, IL). A *p*-value of less than 0.05 was considered as statistically significant. Ethical approval for this study was provided by the Ethical Committee of Siriraj Hospital, Mahidol University, Bangkok, Thailand (Si460/2020) following the tenets of the Declaration of Helsinki. The informed consent was waived by Siriraj Institution Review Board. This study was registered in the Thai Clinical Trials Registry (reg. no. TCTR20200602002, registration date June 2, 2020).

## Results

### Demographic data

Over the ten-year study period, a total of 414 cases involving 416 eyes which had all types of endophthalmitis were identified. Forty eyes of 38 patients with EE were included in this study, which was accounted for 9.18% of the endophthalmitis patients. The mean age was 42.0 ± 21.4 years (range: 2–76 years). The age distribution was somewhat biphasic, with the majority of cases involving people over 21 years old, and with a slightly increasing trend from ages 0–10. The peak frequency was at age 41–50 years. The patients were 63.2% male. The most common associated medical conditions were diabetes mellitus (n = 16, 42.1%), followed by hypertension (n = 6, 15.8%), cirrhosis (n = 4, 10.5%), pulmonary tuberculosis (n = 3, 7.9%), and HIV infection (n = 2, 5.26%). Smoking (n = 11, 28.9%) and alcohol intake (n = 11, 28.9%) were reported, while none involving intravenous drug use was found. Underlying ocular conditions included diabetic retinopathy (n = 5, 12.5%) and glaucoma (n = 1, 2.5%).

### Clinical features

The mean duration from the onset of symptoms to being referred to ophthalmology service was 11.4 ± 11.7 days (range: 1–59 days). The mean follow-up time was 4.8 ± 2.2 years, with a median of four years, and a range from 22 days to 12 years. Treatments prior to presentation at our clinic included any route of antibiotics (n = 19, 47.5%) and steroids (n = 14, 35%). The mean initial best-corrected visual acuity (BCVA) was 2.74 ± 1.30 LogMAR with more than half of our patients (n = 20) having had VA inferior to HM. Our ocular findings included: ocular hypertension (n = 7, 17.5%), presence of RAPD (n = 11, 27.5%), hypopyon (n = 22, 55%), vitreous haze grading ≥ 3 (n = 33, 89.2%), and presence of abnormal retinal lesion (n = 12, 30%).

### Causative organisms and sources of infection

Microbiological isolation was obtained from both ocular and systemic specimens, including aqueous, vitreous, ocular soft tissue, hemoculture, and non-ocular body fluids (pus, cerebrospinal fluid, sputum, or urine). Causative organisms were identified in 27 patients (71.1% culture-proven rate). Identifiable methods included: vitreous (n = 16), hemoculture (n = 12), non-ocular body fluids (n = 6), aqueous (n = 5), and ocular tissue (n = 3). Among our culture-proven cases, gram-negative bacteria were isolated in 13 cases (48.1%), gram-positive bacteria in 11 cases (40.7%), fungus in three cases (11.1%), and *Mycobacterium spp.* in two cases (7.4%). The most common pathogens were *Klebsiella pneumoniae* (n = 7, 25.9%), followed by *Streptococcus spp.* (n = 4, 14.8%), and *Staphylococcus spp*. (n = 4, 14.8%), consecutively. Mixed organisms were identified in two cases. *Staphylococcus spp*. and *Penicillium spp.* were identified as part of the mixed pathogens in each case. From all the EE cases, identifiable infectious sources were present in 19 of them (50%). Among the patients with identifiable infectious sources, the oro-gastrointestinal system was the most responsible one (n = 5, 26.3%), followed by soft tissue and the musculoskeletal system (n = 4, 21.1%). Three cases (15.8%) involved multiple, active sources of infection. For the cases with multiple infection sites, pulmonary infection (n = 4, 21.1%) was the most common organ involved, followed by liver abscess (n = 3, 15.8%). Among the four cases of pulmonary infection, the isolated pathogens were *K. pneumoniae* in two cases and *M. tuberculosis* in another two. Both cases of tuberculous EE were HIV-infected individual. The second most-common site of infection was liver abscess, which were all caused by *K. pneumoniae.* For soft tissue and musculoskeletal infection, the isolated pathogens were both gram-positive bacteria and gram-negative bacteria (Supplementary table).

### Treatment modalities

Medical treatments included antibiotics by topical (n = 39, 97.5%), systemic (intravenous, oral) (n = 33, 86.8%), and intraocular (n = 34, 85%). All cases were initially treated with systemic (intravenous, oral) and/or intravitreal antibiotics. Thirteen patients with unidentified pathogens were empirically treated with a combination of antibiotics, including vancomycin (n = 13, 100%), amikacin (n = 8, 61.5%), ceftazidime (n = 6, 46.2%), clindamycin (n = 1, 7.7%), and cefotaxime (n = 1, 7.7%). Antibiotics were adjusted according to the identified pathogens and the recommendation by the infectious unit (Supplementary table). An oral steroid (prednisolone 20–40 mg/day) was prescribed for six patients. Five patients received after the infection had subsided and there was no growth from culture. In one case, the patient received as a continuation from the previous treatment. A topical steroid was also prescribed. (n = 28, 70%). Ophthalmologic surgeries included pars plana vitrectomy (PPV) (n = 13, 32.5%), enucleation (n = 9, 22.5%), exenteration (n = 1, 2.5%), and orbitotomy (n = 1, 2.5%). Among 13 eyes that underwent PPV, five (38.5%) had PPV twice, and eight (61.5%) had PPV once. The purposes of PPV were for treatment of infection (n = 5, 12.5%) and both treatment of infection and repaired rhegmatogenous retinal detachment (RRD) (n = 7, 17.5%). One eye developed an iatrogenic tear-posterior capsule during vitreous tapping, which required PPV and a lensectomy afterward. The average time from presentation to the first PPV was 10.69 ± 10.09 days (rang: 0–51), with nine out of 13 eyes (69.2%) having PPV within the first week.

### Early outcomes and complications

The mean final BCVA at one month in globe-preserved eyes was 3.11 ± 1.79 LogMAR with 46.7% (n = 14) worse than HM. There was no significant difference between the initial and final VA of the globe-preserving eyes. Ten eyes (25%) were surgically removed, including enucleation in nine and exenteration in the other. The reasons for eye removal were uncontrolled infection in eight eyes (10.3 ± 7.7 days after presentation, range: 1–17 days), and painful blind eye in two eyes (at 50 and 53 days after presentation). Ocular complications presented in 35 eyes (87.5%). The most common ocular complications were ocular hypertension (n = 22, 55.0%), retinal detachment (17, n = 42.5%), panophthalmitis (n = 10, 25.0%), cataracts (n = 8, 20.0%), and phthisis bulbi (n = 7, 17.5%), consecutively. All eyes with ocular hypertension were treated with anti-glaucoma medications, while two eyes underwent trans-scleral cyclophotocoagulation. Among 17 eyes with retinal detachment, the majority had RRD, except in one case with brucellosis in which the patient had exudative retinal detachment. Eight patients (9 eyes) developed RRD at presentation while the rests of them developed RRD during the follow up visits. Seven of 17 eyes with RRD (41.2%) underwent PPV: six eyes with silicone-oil infusion, and one with gas (sulfur hexafluoride; SF6) infusion. Scleral buckling was performed on two eyes. Re-detached RRD occurred in three eyes. Both patients with mycobacterium tuberculous endophthalmitis developed panophthalmitis—one with an orbital abscess, which required exenteration. Another individual developed a scleral abscess in the fellow eye, two months after presentation of the initial eye. The mean duration of inpatient hospitalization was 25.38 ± 31.9 days (range: 7–212 days). Among all of our EE cases, only one patient with *Enterococcus hirae* EE died from a new episode of *E.Coli* peritonitis, with sepsis at six months after the onset of EE.

### Visual prognostic factors

An early post-treatment outcomes at one month was used for analysis. The factors correlations among visual outcome was analyzed as shown in Table 1. The VA at the final follow-up was divided into two groups: equal or better than HM as a favorable outcome, and worse than HM as an unfavorable outcome. The surgically removed eyes were considered as VA of no-light perception. In the univariate analysis, poor initial VA (*p* 0.001), IVT antibiotic use (*p* 0.029), systemic steroid use (*p* 0.029), and PPV (*p* 0.015) significantly correlated with an unfavorable visual outcome. However, as confirmed in the multivariate model, a poor initial VA (worse than HM) was the only significant prognostic factor associated with an unfavorable visual outcome (*p* 0.12, Adjusted OR 10.20, 95% CI 1.65–62.96). The odd ratio of intravitreal antibiotics used was not available, due to the zero value in the 4 × 4 analysis table, so it was not included in the multivariate analysis.

**Table 1:** Prognostic factors associated with unfavorable early post-treatment visual outcome.

Factors	OR*	95% CI^†^	*p*-value	Adjusted OR	95% CI	*p*-value
Diabetes mellitus	1.52	(0.39, 5.91)	0.512			
Time to presentation > 14 days	1.23	(0.29, 5.18)	1.000			
Initial VA^‡^ < HM^§^	12.28	(2.57,58.59)	0.001	10.2	(1.65, 62.96)	.012
Vitreous haze > grade 2	4.55	(50.0, 0.43)	0.296			
Identified organism	2.65	(0.65, 10.75)	0.289			
Gram negative bacteria	1.78	(0.28, 11.12)	0.659			
IVT^¶^ antibiotics use	NA^?^	NA	0.029			
IV** Antibiotics use	3.00	(0.69,13.12)	0.263			
Systemic steroid use	0.10	(0.01, 0.92)	0.029	0.092	(0.007, 1.28)	0.076
Vitrectomy	0.16	(0.04, 0.67)	0.015	0.331	(0.58, 1.89)	0.215

*OR odd ratio, †*CI* confidence interval, ‡*VA* visual acuity, §*HM* hand motion, ¶*IVT* intravitreal, ?*NA* not applicable, **IV intravenous.

### Long-term outcomes at ten years

Only five patients visited our clinic regularly for ten or more years. Among the residual 33 patients, some of them were referred for follow-up treatment at primary care hospitals, and some of them were lost to follow-up. We do not know the mortality rate of these patients; they were relatively healthy during their last follow-up visit at Siriraj Hospital, except for one patient who experienced another episode of sepsis and refused hospitalization. One patient with Klebsiella EE had regained his vision dramatically from HM at discharge to 6/7.5 after ten years. He also had exotropia, which required lateral-rectus muscle recession. Two patients had a declination in their VA at the end of the study. The first patient had a good VA of 6/12 at discharge, but during the follow-up periods, his vision deteriorated to 6/24 because of corneal decompensation. This patient was infected with a combination of *Prevotella spp*. and *Penicillium spp*. Another patient with Klebsiella EE developed RRD, with re-detachment of the retina beneath silicone oil. His vision decreased from 6/96 to HM. The last two patients had a poor initial VA without visual improvement after treatment. Both of them developed phthisis bulbi. One ended up with some perception of light, while the other had no light perception at all. One of them was diagnosed with *Klebsiella pneumoniae* EE.

## Discussion

The long-term visual outcome of EE patients in our study was varied. Immediate post-treatment VA could not predict their final VA at ten years. We had one patient who regained his vision to nearly normal. On the other hand, we also had patients who had visual deterioration related to the development of long-term complications. The relationship between the disease severity and the type of organisms was inconclusive. Our patients who suffered from Klebsiella EE had both good and bad VA at their ten-year follow up. Previous reports have found that most Klebsiella EE patients had a poor visual prognosis, except for the patients who initially had a VA better than HM^8,9^. It seems that patients who had a poor initial VA at presentation tended to continue to have a poor visual outcome. We also found a strong correlation between a poor initial VA and poor early post-treatment visual outcomes, according to the multivariate analysis in this study. Unfortunately, we had a limited number of patients who continued to follow up at our clinic for at least ten years. So, we have been unable to calculate the predictive factors of good or poor long-term visual outcomes. The patient who had good vision at ten years in his follow-up visit had initially suffered from *Klebsiella pneumoniae* pneumonia with meningoencephalitis. He was treated with amoxicillin and clavulanic acid infusion, then meropenem and ceftriaxone in the end. About a week after the onset of systemic infection, he developed endophthalmitis. His initial VA was light perception with whitish plaque over the retina and dense vitritis. He was treated with an intravitreal injection twice (the first injection was ceftazidime and the second was vancomycin plus amikacin). After his infection had subsided for two months, he received PPV to clear the vitreous opacity. He was found to have an epiretinal membrane and shallow peripheral tractional retinal detachment, without macular involvement intraoperatively. Two months later, a second PPV combined with cataract extraction were done to remove significant epiretinal membrane and cataract. His vision improved to 6/48. He developed a macular edema afterward, which responded well to intravitreal bevacizumab. He gained his vision gradually. It took about 1.5 years to regain his vision to 6/7.5. Despite his poor initial VA and the fact that he did not receive an early vitrectomy, he had a wonderful long-term visual outcome. This case is exceptional. Teerittikul et al*.* reported organisms found in EE patients at Siriraj Hospital between 2003 and 2014. Their study period overlapped with that of the present study. The number of EE patients was double in their study, and there were differences involving the common pathogenic bacteria between their study and ours. They found that, after 2011, *Klebsiella spp*., which was formerly known as the most common cause of EE in Asia, was no longer the most common organism in our hospital. *Streptococcus spp*., as in most reports from Europe and the US, was primarily responsible for EE between 2011 and 2014^10,11^. In Thailand, a report by Silpa-archa et al*.* at Rajavithi Hospital showed no increasing trends of gram-positive bacteria between 2005 and 2015^4^. A report from Japan which collected the data of EE patients from 2009 to 2014 also found a four-fold rise of *Staphylococcus aureus* EE, when compared to a published paper in 2001^12,13^. Interestingly, *Klebsiella spp*. showed rising trends in a report from Australia^14^. These organisms’ switching trends have no clear explanation. Another interesting observation found in this study is a high rate of RRD. A total of 16 eyes (40%) had RRD throughout the study period. We collected the data not only at presentation but also upon their long-term follow up visits. The rate of RRD at presentation including development of RRD within 14 days after initial presentation were 8 patients (9 eyes, 22.5%). From previous reports, rate of RRD in endogenous endophthalmitis varied from 4.9% to 26.67%^4,15–18^. Virulence of organism and diabetes were previously reported as possible risk factors of developing RRD in exogenous endophthalmitis^19,20^. Wang et al*.* reported that aphakia and presence of posterior synechiae were associated with the development of RD, but they included both exogenous and endogenous endophthalmitis in their study^15^. In our study, 7 of 16 eyes (43.75%) which developed RRD had hypopyon at presentation and 6 of 15 patients (40%) had diabetes.

### Limitations

This study was a retrospective one aimed at evaluating visual outcomes ten years after patients were diagnosed with EE, so we had to retrospect to the years 2001–2011. Data collection from these medical records, which were 10 to 20 years old, were probably out of date, and some of the information may no longer be useful or applicable. Incomplete data is also a limitation of a retrospective study, which may cause distortions in the statistical calculations. A small number of patients at the end of this study resulted in inadequate information for statistical calculations.

## Conclusions

The long-term visual prognosis and outcomes of EE are mysterious. A lack of information can result in unpredictable long-term visual outcomes. Most of the patients who had a poor initial VA ended up with a poor early post-treatment visual outcome. Complications may play an important role in an individual’s long-term visual prognosis. Factors associated with long-term visual prognosis need to be evaluated in the future.

## Supplementary Information

Supplementary Information.

## Data Availability

The datasets used and/or analyzed during the current study are available from the corresponding author on reasonable request.
