# Supplementary material for: Visual outcome of endogenous endophthalmitis in Thailand
Source: Sci Rep. 2021 Jul 12;11:14313. doi: 10.1038/s41598-021-93730-7 (PMC8275623; doi:10.1038/s41598-021-93730-7)
Supplement: Supplementary file 1 — Supplementary Information. [file 41598_2021_93730_MOESM1_ESM.docx]

**Supplementary table** Summarization of identifiable pathogens and specific treatments in endogenous endophthalmitis

| Case | Organism | Primary source of infection | Pathogen identifiable | | | | | Specific treatments | | |
| --- | --- | --- | --- | --- | --- | --- | --- | --- | --- | --- |
|  |  |  | AQ^*^ | VT^†^ | HC^‡^ | Body fluid | Ocular tissue | Intravitreous | Systemic | |
|  |  |  |  |  |  |  |  |  | Intravenous | Oral |
| 4 | Staphylococcus spp.^§^ | soft tissue infection | NA^¶^ | + GS^?^ | + | + Pus CS^**^ | NA | vancomycin | amoxycillin/ clavulanate | clindamycin |
| 7 | Klebsiella pneumoniae | unidentified | + GS | + GS | + | NA | NA | ceftazidime | ceftriaxone | - |
| 10 | Pseudomonas aeruginosa | soft tissue infection | NA | ­- | + | + Pus CS | NA | ceftazidime, amikacin | piperacillin-tazobactam, ciprofloxacin, ceftazidime, amikacin | ciprofloxacin |
| 12 | Staphylococcus+ E.coli | abscess at buttock | - | - | - | + Pus CS | NA | vancomycin, ceftazidime, amikacin, clindamycin | ciprofloxacin plus metronidazole | ciprofloxacin, metronidazole |
| 13 | Penicillium+ Prevotella | unidentified | + CS | - | NA | NA | NA | fluconazole, clindamycin | voriconazole | itraconazole, levofloxacin |
| 14 | Streptococcus spp. | unidentified | - | + CS | NA | NA | - | vancomycin, ceftazidime | vancomycin, ceftazidime | ofloxacin |
| 15 | Enterococcus hirae | unidentified | + PCR^††^ | + PCR | + | NA | NA | ceftazidime | imipenem/ cilastatin, ampicillin plus gentamicin | levofloxacin |
|  | Enterococcus hirae | unidentified | NA | + PCR | + | NA | NA | ceftazidime | imipenem/ cilastatin, ampicillin plus gentamicin | levofloxacin |
| 16 | Klebsiella pneumoniae | liver abscess | NA | + CS | NA | NA | NA | - | ceftazidime | ofloxacin |
| 17 | Klebsiella pneumoniae | urinary tract infection | NA | - | + | + UC^‡‡^ | - | ceftazidime | ceftazidime, piperacillin/ tazobactam, imipenem/ cilastatin | - |
| 18 | Enterobacter | unidentified | NA | + PCR | - | NA | NA | ceftazidime, amikacin | - | ofloxacin |
| 19 | Acid fast bacilli | pulmonary TB^§§^ | NA | NA | NA | NA | + Pathology | - | amikacin | isoniazid, rifampicin, ethambutol, levofloxacin, clarithromycin |
| 20 | Brucella | Brucellosis | NA | + PCR | + | NA | NA | ceftazidime | amikacin | ciprofloxacin, doxycycline |
| 21 | Aeromonas hydrophila | infectious diarrhea | NA | + CS | NA | NA | NA | ceftazidime | piperacillin/ tazobactam, ciprofloxacin plus metronidazole | ciprofloxacin, metronidazole |
| 22 | Cellulosimicrobium | unidentified | NA | + PCR | NA | NA | NA | vancomycin, amikacin | - | ciprofloxacin |
| 24 | Streptococcus spp. | soft tissue infection | NA | + PCR | - | + Pus CS | NA | vancomycin, ceftazidime | cefazolin | ofloxacin |
| 25 | Klebsiella pneumoniae | acute cholecystitis | NA | + PCR | - | - | NA | ceftazidime | meropenem | moxifloxacin |
| 26 | Klebsiella pneumoniae | liver abscess, pneumonia | NA | + GS | + | - | NA | ceftazidime, amikacin | ceftriaxone, ceftazidime | levofloxacin |
| 27 | Staphylococcus spp. | acute pyelonephritis | - | NA | + | - | NA | vancomycin, ceftazidime | vancomycin, ceftazidime | - |
| 28 | Streptococcus spp. | unidentified | + PCR | - | NA | NA | NA | vancomycin, ceftazidime | cefazolin, amoxycillin/ clavulanate | levofloxacin |
| 29 | Klebsiella pneumoniae | meningitis, liver abscess, pneumonia | - | NA | + | + CSF^¶¶^, sputum CS | NA | amikacin | amoxycillin/ clavulanate, meropenem, ceftriaxone | - |
| 30 | Klebsiella pneumoniae | splenic abscess | NA | + CS | - | - | NA | ceftazidime | ceftazidime, imipenem/ cilastin, ceftriaxone | levofloxacin |
| 31 | Aspergillus | unidentified | NA | - | NA | NA | + Pathology | - | amphotericin | - |
| 32 | Streptococcus spp. | unidentified | NA | + CS | - | NA | NA | vancomycin, ceftazidime | vancomycin, ceftazidime | Ofloxacin |
| 33 | Acid fast bacilli | pulmonary TB | NA | - | NA | NA | + Pathology | amikacin | ciprofloxacin | isoniazid, rifampicin, ciprofloxacin, clarithromycin |
| 36 | Aspergillus | unidentified | NA | + CS | NA | NA | NA | amphotericin | amphotericin | - |
| 38 | Staphylococcus spp. | unidentified | NA | NA | + | NA | NA | - | ceftriaxone | ofloxacin |

* AQ = aqueous, † VT = vitreous, ‡ HC = hemoculture, § SPP = species, ¶ NA = not applicable, ? GS = gram stain, ** CS = culture, †† PCR = polymerase chain reaction, ‡‡ UC = urine culture, §§ TB = tuberculosis, ¶ ¶ CSF = cerebrospinal fluid
